# 1-Ethyl-3,3-di­methyl­spiro­[indoline-2,8′-phenaleno[1,9-*fg*]chromene]

**DOI:** 10.1107/S2414314624001378

**Published:** 2024-02-13

**Authors:** Xiaoming Zhu., Zhen Jiang, Zhiqiang Liu

**Affiliations:** a State Key Laboratory of Crystal Materials Shandong University,Jinan 250100 People’s Republic of China; University of Aberdeen, United Kingdom

**Keywords:** crystal structure, spiro­pyran, pyrene, packing

## Abstract

The title pyrene-fused spiro­pyran derivative, C_30_H_25_NO, crystallizes with two mol­ecules in the asymmetric unit with dihedral angles between their fused-ring sub units of 76.20 (8) and 89.38 (9)°. In the crystal, weak C—H⋯π inter­actions link the mol­ecules into a three-dimensional network.

## Abstract 2

Both mol­ecules in the asymmetric unit of the title compound are twisted. In the crystal, weak C—H⋯π inter­actions link the mol­ecules into a three-dimensional network.

## Graphical abstract

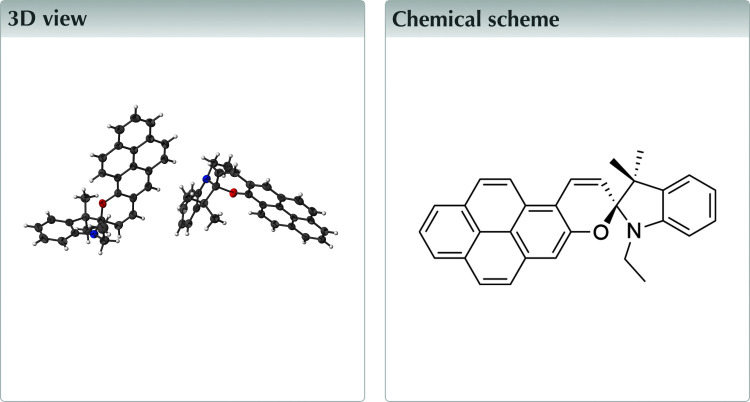

## Structure description

As a photochromic material, spiro­pyran has emerged as a platform for developing new types of dynamic materials, which can respond with reversible isomerization to different stimuli such as solvents, metal ions, acids and bases and temperature (Klajn, 2014 ▸; Kozlenko *et al.*, 2023 ▸). Many inter­esting strategies have been applied over the past decades to construct a spiro­pyran-based probe with particular purposes (Das *et al.*, 2023 ▸; He *et al.*, 2021 ▸). As a classical polycyclic aromatic hydro­carbon and promising chromophore, pyrene is often adapted to build or extend fluorescent materials (Yao *et al.*, 2018 ▸; Zhou *et al.*, 2011 ▸). Herein, we describe the synthesis and structure of the title compound, which is new derivative of spiro­pyran featuring pyrene substitution.

**Figure 1:**
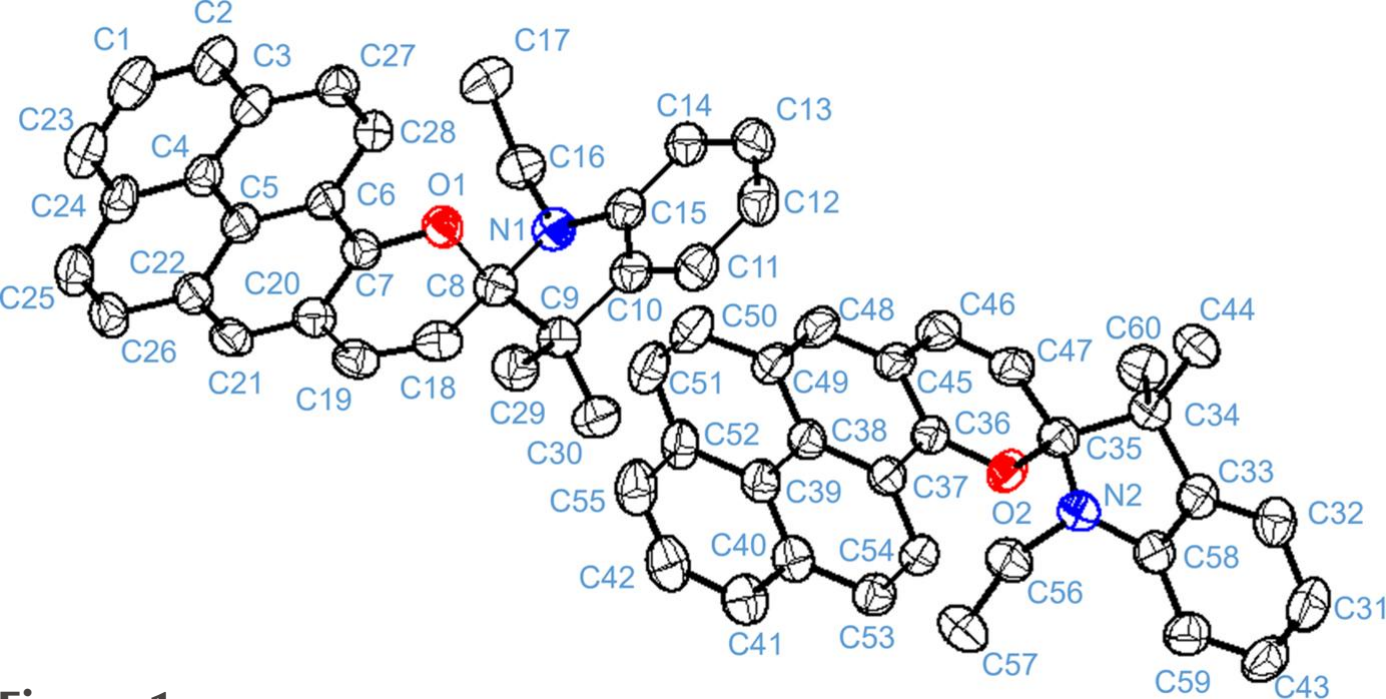
The mol­ecular structure of the title compound showing 50% displacement ellipsoids. H atoms omitted for clarity.

The title compound crystallizes in the uncommon space group *Fdd*2 with two mol­ecules (*A* containing C1 and *B* containing C31) in the asymmetric unit (Fig. 1 ▸). In each mol­ecule, the phenyl group of the indole moiety is nearly perpendicular to the chromene moiety [dihedral angles for mol­ecules *A* and *B* are 76.20 (8) and 89.38 (9)°, respectively]. The central *sp^3^* spiral carbon atoms (C8 in *A* and C35 in *B*) adopt distorted tetra­hedral geometries with the smallest and largest bond angles being C9—C8—N1 = 102.94 (17) and C18—C8—N1 = 114.76 (17)° in *A* and C34—C35—N2 = 103.49 (17) and C34—C35—C47 = 114.32 (18)° in *B*. These *spiro*-carbon atoms are stereogenic (chiral) centres: in the arbitrarily chosen asymmetric unit both have an *R* configuration, but crystal symmetry generates a racemic mixture. The C8—N1—C16—C17 and C35—N2—C56—C57 torsion angles are 82.0 (3) and 81.6 (3)°, respectively.

**Figure 2:**
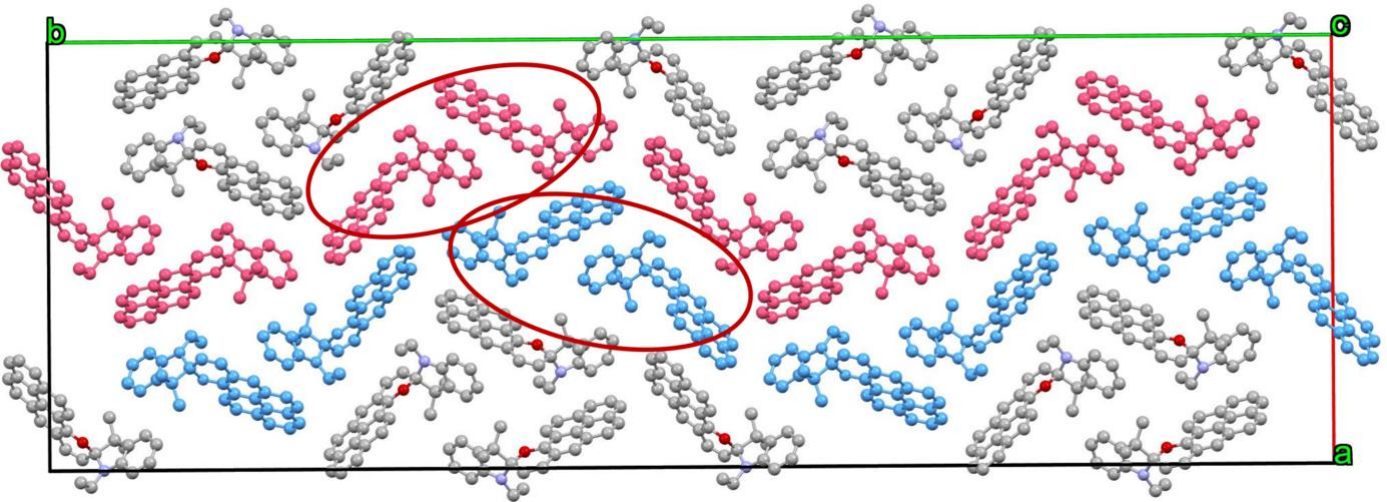
The unit-cell packing viewed down [001].

In the extended structure of the title compound, C—H⋯π inter­actions (Table 1 ▸) link the mol­ecules into a three-dimensional network, which features wave-like chains of mol­ecules propagating along the [010] direction (Fig. 2 ▸).

## Synthesis and crystallization

The synthesis of 2-hy­droxy-1-pyrenecarbaldehyde followed the previously reported procedure (Luong *et al.*, 2020 ▸). Then, 2-hy­droxy-1-pyrenecarbaldehyde and 2,3,3-trimethyl-1-ethyl-indole were added to 20 ml of aceto­nitrile in a Schlenk tube. After heating for 12 h at 85°C, the mixture was cooled to room temperature and the precipitate was recovered by filtration.

Single crystals of the title compound were obtained as pale-yellow plates by slow diffusion of hexane into its chloro­form solution at room temperature. A suitable crystal for data collection was chosen under an optical microscope and quickly coated with high vacuum grease (Dow Corning Corporation) to prevent decomposition.

## Refinement

Crystal data, data collection and structure refinement details are summarized in Table 2 ▸.

## Supplementary Material

Crystal structure: contains datablock(s) I. DOI: 10.1107/S2414314624001378/hb4461sup1.cif

Structure factors: contains datablock(s) I. DOI: 10.1107/S2414314624001378/hb4461Isup2.hkl

CCDC reference: 2109994

Additional supporting information:  crystallographic information; 3D view; checkCIF report

## Figures and Tables

**Table 1:** Hydrogen-bond geometry (Å, °) *Cg*3, *Cg*6, *Cg*7, *Cg*29 and *Cg*31 are the centroids of the C1–C4/C24/C23, C5–C7/C20–C22, C10–C15, C36–C38/C45/C48/C49 and C38/C39/C49–C52 rings, respectively.

*D*—H⋯*A*	*D*—H	H⋯*A*	*D*⋯*A*	*D*—H⋯*A*
C16—H5⋯*Cg*6^i^	0.99	2.91	3.683 (3)	136
C2—H12⋯*Cg*3^ii^	0.95	2.86	3.698 (3)	148
C48—H35⋯*Cg*7	0.95	2.80	3.641 (3)	148
C56—H42⋯*Cg*29^iii^	0.99	2.96	3.856 (2)	151
C59—H47⋯*Cg*31^iii^	0.95	2.98	3.800 (3)	146

Symmetry codes: (i) 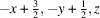; (ii) 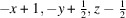; (iii) 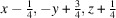.

**Table 2:** Experimental details

Crystal data	
Chemical formula	C_30_H_25_NO
*M*_r_	415.51
Crystal system, space group	Orthorhombic, *Fdd*2
Temperature (K)	150
*a*, *b*, *c* (Å)	27.8745 (9), 83.475 (3), 7.5368 (2)
*V* (Å^3^)	17536.7 (10)
*Z*	32
Radiation type	Cu *K*α
μ (mm^−1^)	0.58
Crystal size (mm)	0.33 × 0.13 × 0.06

Data collection	
Diffractometer	Bruker D8 VENTURE
Absorption correction	Multi-scan (*SADABS*; Krause *et al.*, 2015 ▸)
*T*_min_, *T*_max_	0.833, 0.968
No. of measured, independent and observed [*I* > 2σ(*I*)] reflections	48787, 7883, 7093
*R*_int_	0.039
(sin θ/λ)_max_ (Å^−1^)	0.618

Refinement	
*R*[*F*^2^ > 2σ(*F*^2^)], *wR*(*F*^2^), *S*	0.032, 0.088, 1.05
No. of reflections	7883
No. of parameters	583
No. of restraints	1
H-atom treatment	H-atom parameters constrained
Δρ_max_, Δρ_min_ (e Å^−3^)	0.14, −0.12
Absolute structure	Flack *x* determined using 2576 quotients [(*I*^+^)−(*I*^−^)]/[(*I*^+^)+(*I*^−^)] (Parsons *et al.*, 2013 ▸)
Absolute structure parameter	0.05 (11)

Computer programs: *APEX2* and *SAINT* (Bruker, 2014 ▸), *SHELXS97* and *SHELXTL* (Sheldrick 2008 ▸) and *SHELXL2014/7* (Sheldrick, 2015 ▸).

## full crystallographic data

### 1-Ethyl-3,3-dimethylspiro[indoline-2,8'-phenaleno[1,9-fg]chromene] Crystal data

**Table:**

C_30_H_25_NO	*D*_x_ = 1.259 Mg m^−^^3^
*M**_r_* = 415.51	Cu *K*α radiation, λ = 1.54178 Å
Orthorhombic, *F**d**d*2	Cell parameters from 9930 reflections
*a* = 27.8745 (9) Å	θ = 3.3–72.2°
*b* = 83.475 (3) Å	µ = 0.58 mm^−^^1^
*c* = 7.5368 (2) Å	*T* = 150 K
*V* = 17536.7 (10) Å^3^	Plate, yellow
*Z* = 32	0.33 × 0.13 × 0.06 mm
*F*(000) = 7040	

### 1-Ethyl-3,3-dimethylspiro[indoline-2,8'-phenaleno[1,9-fg]chromene] Data collection

**Table:**

Bruker D8 VENTURE diffractometer	7093 reflections with *I* > 2σ(*I*)
Detector resolution: 8.3 pixels mm^-1^	*R*_int_ = 0.039
φ and ω scans	θ_max_ = 72.3°, θ_min_ = 2.1°
Absorption correction: multi-scan (SADABS; Krause et al., 2015)	*h* = −34→34
*T*_min_ = 0.833, *T*_max_ = 0.968	*k* = −102→102
48787 measured reflections	*l* = −7→9
7883 independent reflections	

### 1-Ethyl-3,3-dimethylspiro[indoline-2,8'-phenaleno[1,9-fg]chromene] Refinement

**Table:**

Refinement on *F*^2^	Hydrogen site location: inferred from neighbouring sites
Least-squares matrix: full	H-atom parameters constrained
*R*[*F*^2^ > 2σ(*F*^2^)] = 0.032	*w* = 1/[σ^2^(*F*_o_^2^) + (0.0516*P*)^2^ + 4.5797*P*] where *P* = (*F*_o_^2^ + 2*F*_c_^2^)/3
*wR*(*F*^2^) = 0.088	(Δ/σ)_max_ = 0.001
*S* = 1.05	Δρ_max_ = 0.14 e Å^−^^3^
7883 reflections	Δρ_min_ = −0.12 e Å^−^^3^
583 parameters	Absolute structure: Flack *x* determined using 2576 quotients [(*I*^+^)-(*I*^-^)]/[(*I*^+^)+(*I*^-^)] (Parsons *et al.*, 2013)
1 restraint	Absolute structure parameter: 0.05 (11)
Primary atom site location: structure-invariant direct methods	

### 1-Ethyl-3,3-dimethylspiro[indoline-2,8'-phenaleno[1,9-fg]chromene] Special details

**Table:**

Geometry. All esds (except the esd in the dihedral angle between two l.s. planes) are estimated using the full covariance matrix. The cell esds are taken into account individually in the estimation of esds in distances, angles and torsion angles; correlations between esds in cell parameters are only used when they are defined by crystal symmetry. An approximate (isotropic) treatment of cell esds is used for estimating esds involving l.s. planes.

### 1-Ethyl-3,3-dimethylspiro[indoline-2,8'-phenaleno[1,9-fg]chromene] Fractional atomic coordinates and isotropic or equivalent isotropic displacement parameters (Å^2^)

**Table:**

	*x*	*y*	*z*	*U*_iso_*/*U*_eq_	
O1	0.68396 (5)	0.27565 (2)	0.2726 (2)	0.0402 (3)	
O2	0.78817 (5)	0.38019 (2)	0.6660 (2)	0.0409 (3)	
N1	0.75013 (6)	0.29216 (2)	0.3061 (3)	0.0406 (4)	
N2	0.72477 (6)	0.39843 (2)	0.6480 (2)	0.0391 (4)	
C1	0.49157 (8)	0.22651 (3)	−0.1132 (4)	0.0526 (6)	
H1	0.4682	0.2226	−0.1943	0.063*	
C2	0.52104 (8)	0.23919 (3)	−0.1621 (4)	0.0466 (5)	
H12	0.5179	0.2438	−0.2767	0.056*	
C3	0.55520 (7)	0.24517 (2)	−0.0444 (3)	0.0389 (5)	
C4	0.56028 (7)	0.23788 (2)	0.1260 (3)	0.0376 (4)	
C5	0.59466 (7)	0.24381 (2)	0.2485 (3)	0.0353 (4)	
C6	0.62375 (7)	0.25712 (2)	0.1999 (3)	0.0351 (4)	
C7	0.65673 (7)	0.26294 (2)	0.3240 (3)	0.0366 (4)	
C8	0.71032 (7)	0.28537 (3)	0.4033 (3)	0.0399 (5)	
C9	0.67930 (7)	0.30043 (3)	0.4504 (3)	0.0412 (5)	
C10	0.69106 (7)	0.31118 (3)	0.2957 (3)	0.0397 (5)	
C11	0.66867 (8)	0.32476 (3)	0.2334 (3)	0.0459 (5)	
H25	0.6396	0.3284	0.2858	0.055*	
C12	0.68919 (9)	0.33315 (3)	0.0921 (4)	0.0488 (5)	
H2	0.6738	0.3425	0.0469	0.059*	
C13	0.73180 (9)	0.32794 (3)	0.0178 (3)	0.0482 (5)	
H3	0.7457	0.3338	−0.0770	0.058*	
C14	0.75482 (8)	0.31420 (3)	0.0791 (3)	0.0445 (5)	
H4	0.7841	0.3107	0.0278	0.053*	
C15	0.73375 (7)	0.30585 (2)	0.2170 (3)	0.0391 (4)	
C16	0.78824 (8)	0.28208 (3)	0.2337 (3)	0.0473 (5)	
H5	0.7957	0.2736	0.3215	0.057*	
H6	0.8174	0.2887	0.2198	0.057*	
C17	0.77745 (10)	0.27406 (3)	0.0559 (4)	0.0604 (7)	
H8	0.7506	0.2666	0.0701	0.091*	
H9	0.8059	0.2682	0.0154	0.091*	
H7	0.7690	0.2823	−0.0318	0.091*	
C18	0.72429 (8)	0.27535 (3)	0.5602 (3)	0.0446 (5)	
H18	0.7511	0.2785	0.6300	0.054*	
C19	0.70026 (8)	0.26220 (3)	0.6043 (3)	0.0438 (5)	
H10	0.7078	0.2569	0.7122	0.053*	
C20	0.66256 (7)	0.25577 (3)	0.4907 (3)	0.0394 (4)	
C21	0.63335 (8)	0.24281 (3)	0.5358 (3)	0.0414 (5)	
H11	0.6370	0.2380	0.6492	0.050*	
C22	0.59920 (7)	0.23676 (2)	0.4196 (3)	0.0391 (5)	
C23	0.49588 (8)	0.21950 (3)	0.0514 (4)	0.0520 (6)	
H15	0.4755	0.2108	0.0817	0.062*	
C24	0.52960 (7)	0.22493 (2)	0.1750 (4)	0.0441 (5)	
C25	0.53434 (8)	0.21828 (3)	0.3484 (4)	0.0485 (6)	
H13	0.5137	0.2098	0.3834	0.058*	
C26	0.56729 (8)	0.22374 (3)	0.4634 (3)	0.0450 (5)	
H14	0.5697	0.2189	0.5771	0.054*	
C27	0.58500 (7)	0.25868 (3)	−0.0873 (3)	0.0405 (5)	
H16	0.5816	0.2637	−0.1999	0.049*	
C28	0.61772 (7)	0.26445 (2)	0.0283 (3)	0.0379 (4)	
H17	0.6369	0.2734	−0.0038	0.045*	
C29	0.62612 (8)	0.29652 (3)	0.4726 (4)	0.0492 (5)	
H21	0.6086	0.3063	0.5046	0.074*	
H20	0.6222	0.2885	0.5667	0.074*	
H19	0.6134	0.2923	0.3609	0.074*	
C30	0.69756 (9)	0.30836 (3)	0.6232 (3)	0.0498 (5)	
H22	0.7323	0.3100	0.6153	0.075*	
H24	0.6903	0.3014	0.7244	0.075*	
H23	0.6816	0.3187	0.6394	0.075*	
C31	0.79572 (10)	0.43889 (3)	0.8270 (4)	0.0552 (6)	
H26	0.8132	0.4481	0.8642	0.066*	
C32	0.81345 (8)	0.42939 (3)	0.6893 (4)	0.0480 (5)	
H29	0.8427	0.4321	0.6317	0.058*	
C33	0.78762 (7)	0.41609 (3)	0.6387 (3)	0.0402 (5)	
C34	0.79542 (7)	0.40448 (3)	0.4869 (3)	0.0401 (5)	
C35	0.76095 (7)	0.39047 (3)	0.5436 (3)	0.0377 (4)	
C36	0.79747 (7)	0.36449 (2)	0.6332 (3)	0.0352 (4)	
C37	0.82225 (6)	0.35612 (2)	0.7649 (3)	0.0346 (4)	
C38	0.83340 (7)	0.33967 (2)	0.7347 (3)	0.0362 (4)	
C39	0.85763 (7)	0.33074 (3)	0.8681 (3)	0.0386 (5)	
C40	0.87026 (7)	0.33803 (3)	1.0307 (3)	0.0417 (5)	
C41	0.89302 (8)	0.32885 (3)	1.1613 (4)	0.0509 (6)	
H39	0.9013	0.3336	1.2718	0.061*	
C42	0.90363 (9)	0.31284 (3)	1.1303 (4)	0.0553 (6)	
H27	0.9195	0.3068	1.2194	0.066*	
C43	0.75289 (10)	0.43503 (3)	0.9099 (4)	0.0546 (6)	
H28	0.7412	0.4417	1.0025	0.066*	
C44	0.77800 (9)	0.41241 (3)	0.3140 (3)	0.0505 (6)	
H31	0.7440	0.4153	0.3256	0.076*	
H30	0.7820	0.4049	0.2151	0.076*	
H32	0.7969	0.4221	0.2913	0.076*	
C45	0.78170 (7)	0.35692 (3)	0.4769 (3)	0.0397 (5)	
C46	0.75138 (8)	0.36604 (3)	0.3590 (3)	0.0447 (5)	
H33	0.7390	0.3611	0.2553	0.054*	
C47	0.74031 (8)	0.38122 (3)	0.3921 (3)	0.0427 (5)	
H34	0.7182	0.3865	0.3157	0.051*	
C48	0.79326 (8)	0.34089 (3)	0.4491 (3)	0.0454 (5)	
H35	0.7833	0.3358	0.3425	0.054*	
C49	0.81906 (7)	0.33215 (3)	0.5736 (3)	0.0416 (5)	
C50	0.83064 (8)	0.31546 (3)	0.5486 (4)	0.0513 (6)	
H41	0.8218	0.3103	0.4411	0.062*	
C51	0.85366 (8)	0.30704 (3)	0.6740 (4)	0.0527 (6)	
H40	0.8605	0.2961	0.6531	0.063*	
C52	0.86823 (7)	0.31413 (3)	0.8381 (4)	0.0450 (5)	
C53	0.85825 (7)	0.35469 (3)	1.0575 (3)	0.0419 (5)	
H36	0.8664	0.3597	1.1667	0.050*	
C54	0.83572 (7)	0.36331 (2)	0.9311 (3)	0.0377 (4)	
H37	0.8287	0.3743	0.9521	0.045*	
C55	0.89149 (8)	0.30561 (3)	0.9725 (4)	0.0538 (6)	
H38	0.8990	0.2946	0.9546	0.065*	
C56	0.68720 (8)	0.38924 (3)	0.7366 (3)	0.0460 (5)	
H43	0.6766	0.3806	0.6555	0.055*	
H42	0.6594	0.3964	0.7568	0.055*	
C57	0.70128 (9)	0.38166 (3)	0.9140 (4)	0.0564 (6)	
H46	0.7249	0.3732	0.8934	0.085*	
H45	0.6727	0.3771	0.9706	0.085*	
H44	0.7152	0.3899	0.9914	0.085*	
C58	0.74486 (7)	0.41221 (2)	0.7245 (3)	0.0390 (4)	
C59	0.72669 (9)	0.42158 (3)	0.8602 (3)	0.0480 (5)	
H47	0.6974	0.4189	0.9174	0.058*	
C60	0.84729 (8)	0.39879 (3)	0.4620 (4)	0.0538 (6)	
H50	0.8675	0.4079	0.4279	0.081*	
H49	0.8484	0.3906	0.3686	0.081*	
H48	0.8591	0.3942	0.5734	0.081*	

### 1-Ethyl-3,3-dimethylspiro[indoline-2,8'-phenaleno[1,9-fg]chromene] Atomic displacement parameters (Å^2^)

**Table:**

	*U*^11^	*U*^22^	*U*^33^	*U*^12^	*U*^13^	*U*^23^
O1	0.0442 (7)	0.0403 (8)	0.0362 (8)	−0.0077 (6)	−0.0033 (6)	0.0017 (6)
O2	0.0530 (8)	0.0330 (7)	0.0368 (8)	0.0030 (6)	−0.0096 (7)	−0.0040 (6)
N1	0.0361 (8)	0.0424 (9)	0.0433 (10)	−0.0014 (7)	−0.0006 (7)	−0.0029 (8)
N2	0.0393 (8)	0.0412 (9)	0.0368 (9)	0.0004 (7)	0.0003 (7)	0.0017 (8)
C1	0.0409 (11)	0.0437 (12)	0.0732 (18)	0.0056 (9)	−0.0102 (11)	−0.0153 (12)
C2	0.0422 (10)	0.0397 (11)	0.0581 (14)	0.0077 (9)	−0.0079 (10)	−0.0104 (10)
C3	0.0372 (9)	0.0338 (10)	0.0458 (12)	0.0078 (8)	−0.0022 (9)	−0.0082 (9)
C4	0.0352 (9)	0.0296 (9)	0.0479 (12)	0.0051 (7)	0.0056 (9)	−0.0047 (9)
C5	0.0350 (9)	0.0301 (9)	0.0408 (11)	0.0064 (7)	0.0050 (8)	−0.0011 (9)
C6	0.0359 (9)	0.0324 (9)	0.0371 (11)	0.0030 (7)	0.0038 (8)	−0.0020 (8)
C7	0.0373 (9)	0.0343 (10)	0.0381 (11)	0.0012 (8)	0.0028 (9)	0.0015 (9)
C8	0.0362 (9)	0.0441 (11)	0.0395 (11)	−0.0042 (8)	−0.0045 (8)	−0.0022 (9)
C9	0.0379 (10)	0.0447 (11)	0.0409 (12)	−0.0028 (8)	0.0000 (9)	−0.0013 (10)
C10	0.0366 (9)	0.0418 (11)	0.0408 (12)	−0.0069 (8)	−0.0027 (9)	−0.0052 (9)
C11	0.0409 (10)	0.0435 (11)	0.0533 (14)	−0.0033 (9)	−0.0022 (10)	−0.0066 (11)
C12	0.0518 (12)	0.0407 (11)	0.0539 (14)	−0.0069 (10)	−0.0107 (11)	0.0021 (11)
C13	0.0549 (12)	0.0479 (13)	0.0420 (13)	−0.0167 (10)	−0.0021 (10)	0.0003 (10)
C14	0.0426 (10)	0.0453 (12)	0.0456 (13)	−0.0095 (9)	0.0020 (10)	−0.0059 (10)
C15	0.0384 (9)	0.0394 (10)	0.0396 (11)	−0.0082 (8)	−0.0031 (9)	−0.0062 (9)
C16	0.0431 (10)	0.0516 (13)	0.0473 (13)	0.0034 (9)	0.0010 (10)	−0.0038 (11)
C17	0.0667 (15)	0.0575 (15)	0.0572 (16)	0.0075 (12)	0.0036 (13)	−0.0157 (13)
C18	0.0408 (10)	0.0552 (13)	0.0379 (12)	0.0033 (9)	−0.0069 (9)	−0.0018 (10)
C19	0.0453 (11)	0.0504 (12)	0.0356 (11)	0.0057 (9)	−0.0024 (9)	0.0045 (10)
C20	0.0413 (10)	0.0410 (11)	0.0359 (11)	0.0062 (8)	0.0009 (8)	0.0015 (9)
C21	0.0456 (10)	0.0408 (11)	0.0379 (11)	0.0091 (9)	0.0076 (9)	0.0070 (9)
C22	0.0396 (9)	0.0332 (10)	0.0445 (12)	0.0068 (8)	0.0082 (9)	0.0021 (9)
C23	0.0386 (10)	0.0349 (11)	0.0825 (18)	−0.0009 (9)	0.0028 (11)	−0.0110 (12)
C24	0.0398 (10)	0.0288 (9)	0.0637 (15)	0.0050 (8)	0.0079 (10)	−0.0064 (10)
C25	0.0429 (10)	0.0330 (10)	0.0697 (17)	0.0016 (9)	0.0133 (11)	0.0007 (11)
C26	0.0470 (11)	0.0348 (10)	0.0532 (14)	0.0070 (9)	0.0139 (10)	0.0080 (10)
C27	0.0444 (10)	0.0384 (10)	0.0388 (11)	0.0055 (8)	−0.0018 (9)	−0.0008 (9)
C28	0.0402 (9)	0.0343 (10)	0.0392 (11)	−0.0012 (8)	0.0007 (8)	0.0002 (9)
C29	0.0409 (11)	0.0507 (12)	0.0560 (14)	−0.0003 (9)	0.0064 (10)	0.0017 (11)
C30	0.0508 (12)	0.0540 (13)	0.0447 (13)	−0.0018 (10)	0.0016 (10)	−0.0088 (11)
C31	0.0676 (15)	0.0364 (11)	0.0616 (16)	0.0046 (11)	−0.0202 (13)	−0.0038 (11)
C32	0.0497 (11)	0.0396 (11)	0.0546 (14)	0.0016 (9)	−0.0090 (11)	0.0040 (10)
C33	0.0411 (10)	0.0368 (10)	0.0426 (12)	0.0056 (8)	−0.0075 (9)	0.0025 (9)
C34	0.0406 (10)	0.0413 (11)	0.0382 (11)	0.0000 (8)	0.0006 (9)	0.0017 (9)
C35	0.0401 (9)	0.0388 (10)	0.0342 (11)	0.0022 (8)	−0.0034 (8)	0.0024 (9)
C36	0.0364 (9)	0.0332 (10)	0.0359 (11)	−0.0029 (7)	0.0029 (8)	−0.0023 (9)
C37	0.0322 (8)	0.0348 (10)	0.0369 (11)	−0.0036 (7)	0.0040 (8)	−0.0023 (8)
C38	0.0318 (8)	0.0347 (10)	0.0420 (11)	−0.0030 (7)	0.0062 (8)	−0.0003 (9)
C39	0.0306 (8)	0.0367 (10)	0.0484 (12)	−0.0024 (7)	0.0063 (9)	0.0009 (9)
C40	0.0340 (9)	0.0414 (11)	0.0497 (13)	−0.0007 (8)	0.0002 (9)	0.0046 (10)
C41	0.0477 (11)	0.0499 (13)	0.0551 (14)	0.0026 (10)	−0.0062 (11)	0.0060 (12)
C42	0.0487 (12)	0.0481 (13)	0.0692 (17)	0.0067 (10)	−0.0045 (12)	0.0155 (13)
C43	0.0765 (16)	0.0398 (12)	0.0476 (13)	0.0159 (11)	−0.0071 (13)	−0.0072 (11)
C44	0.0549 (12)	0.0541 (13)	0.0426 (13)	−0.0053 (11)	0.0020 (11)	0.0103 (11)
C45	0.0414 (10)	0.0411 (11)	0.0365 (11)	−0.0073 (8)	0.0014 (9)	−0.0059 (9)
C46	0.0482 (11)	0.0501 (13)	0.0359 (11)	−0.0056 (9)	−0.0047 (9)	−0.0061 (10)
C47	0.0443 (11)	0.0505 (12)	0.0333 (11)	−0.0025 (9)	−0.0041 (9)	0.0006 (10)
C48	0.0489 (11)	0.0442 (11)	0.0430 (12)	−0.0054 (9)	−0.0022 (10)	−0.0139 (10)
C49	0.0390 (10)	0.0375 (10)	0.0483 (13)	−0.0036 (8)	0.0042 (9)	−0.0103 (10)
C50	0.0482 (11)	0.0421 (12)	0.0635 (16)	−0.0024 (10)	0.0028 (12)	−0.0174 (11)
C51	0.0449 (11)	0.0344 (11)	0.0788 (19)	0.0003 (9)	0.0082 (12)	−0.0107 (12)
C52	0.0342 (9)	0.0355 (10)	0.0653 (15)	0.0002 (8)	0.0090 (10)	0.0014 (10)
C53	0.0442 (10)	0.0416 (11)	0.0400 (12)	0.0005 (9)	−0.0040 (9)	−0.0010 (10)
C54	0.0409 (10)	0.0336 (10)	0.0387 (11)	0.0001 (8)	0.0001 (9)	−0.0024 (9)
C55	0.0431 (11)	0.0385 (12)	0.0798 (19)	0.0056 (9)	0.0060 (12)	0.0058 (13)
C56	0.0408 (10)	0.0538 (13)	0.0432 (12)	−0.0029 (9)	0.0010 (9)	0.0037 (11)
C57	0.0579 (13)	0.0651 (16)	0.0464 (14)	−0.0075 (12)	0.0024 (11)	0.0138 (12)
C58	0.0425 (10)	0.0369 (10)	0.0374 (11)	0.0062 (8)	−0.0043 (9)	0.0035 (9)
C59	0.0548 (12)	0.0462 (12)	0.0429 (12)	0.0121 (10)	−0.0006 (10)	−0.0001 (10)
C60	0.0438 (11)	0.0567 (14)	0.0609 (16)	0.0014 (10)	0.0070 (11)	−0.0025 (12)

### 1-Ethyl-3,3-dimethylspiro[indoline-2,8'-phenaleno[1,9-fg]chromene] Geometric parameters (Å, º)

**Table:**

O1—C7	1.360 (2)	C29—H20	0.9800
O1—C8	1.473 (3)	C29—H19	0.9800
O2—C36	1.359 (2)	C30—H22	0.9800
O2—C35	1.471 (2)	C30—H24	0.9800
N1—C15	1.402 (3)	C30—H23	0.9800
N1—C8	1.445 (3)	C31—C43	1.386 (4)
N1—C16	1.461 (3)	C31—C32	1.396 (4)
N2—C58	1.403 (3)	C31—H26	0.9500
N2—C35	1.441 (3)	C32—C33	1.378 (3)
N2—C56	1.460 (3)	C32—H29	0.9500
C1—C23	1.377 (4)	C33—C58	1.394 (3)
C1—C2	1.389 (4)	C33—C34	1.514 (3)
C1—H1	0.9500	C34—C60	1.534 (3)
C2—C3	1.394 (3)	C34—C44	1.540 (3)
C2—H12	0.9500	C34—C35	1.573 (3)
C3—C4	1.428 (3)	C35—C47	1.494 (3)
C3—C27	1.438 (3)	C36—C37	1.397 (3)
C4—C5	1.420 (3)	C36—C45	1.407 (3)
C4—C24	1.427 (3)	C37—C38	1.426 (3)
C5—C6	1.423 (3)	C37—C54	1.439 (3)
C5—C22	1.423 (3)	C38—C49	1.424 (3)
C6—C7	1.398 (3)	C38—C39	1.422 (3)
C6—C28	1.441 (3)	C39—C40	1.413 (3)
C7—C20	1.401 (3)	C39—C52	1.435 (3)
C8—C18	1.500 (3)	C40—C41	1.400 (3)
C8—C9	1.566 (3)	C40—C53	1.445 (3)
C9—C10	1.507 (3)	C41—C42	1.389 (4)
C9—C29	1.527 (3)	C41—H39	0.9500
C9—C30	1.547 (3)	C42—C55	1.376 (4)
C10—C11	1.377 (3)	C42—H27	0.9500
C10—C15	1.402 (3)	C43—C59	1.391 (4)
C11—C12	1.397 (4)	C43—H28	0.9500
C11—H25	0.9500	C44—H31	0.9800
C12—C13	1.383 (4)	C44—H30	0.9800
C12—H2	0.9500	C44—H32	0.9800
C13—C14	1.393 (3)	C45—C48	1.392 (3)
C13—H3	0.9500	C45—C46	1.443 (3)
C14—C15	1.382 (3)	C46—C47	1.328 (3)
C14—H4	0.9500	C46—H33	0.9500
C16—C17	1.528 (4)	C47—H34	0.9500
C16—H5	0.9900	C48—C49	1.389 (3)
C16—H6	0.9900	C48—H35	0.9500
C17—H8	0.9800	C49—C50	1.443 (3)
C17—H9	0.9800	C50—C51	1.341 (4)
C17—H7	0.9800	C50—H41	0.9500
C18—C19	1.328 (3)	C51—C52	1.431 (4)
C18—H18	0.9500	C51—H40	0.9500
C19—C20	1.458 (3)	C52—C55	1.397 (4)
C19—H10	0.9500	C53—C54	1.349 (3)
C20—C21	1.396 (3)	C53—H36	0.9500
C21—C22	1.388 (3)	C54—H37	0.9500
C21—H11	0.9500	C55—H38	0.9500
C22—C26	1.443 (3)	C56—C57	1.530 (4)
C23—C24	1.398 (4)	C56—H43	0.9900
C23—H15	0.9500	C56—H42	0.9900
C24—C25	1.426 (4)	C57—H46	0.9800
C25—C26	1.342 (4)	C57—H45	0.9800
C25—H13	0.9500	C57—H44	0.9800
C26—H14	0.9500	C58—C59	1.384 (3)
C27—C28	1.350 (3)	C59—H47	0.9500
C27—H16	0.9500	C60—H50	0.9800
C28—H17	0.9500	C60—H49	0.9800
C29—H21	0.9800	C60—H48	0.9800
			
C7—O1—C8	121.17 (16)	H22—C30—H24	109.5
C36—O2—C35	123.18 (16)	C9—C30—H23	109.5
C15—N1—C8	108.19 (16)	H22—C30—H23	109.5
C15—N1—C16	121.8 (2)	H24—C30—H23	109.5
C8—N1—C16	121.46 (18)	C43—C31—C32	120.5 (2)
C58—N2—C35	108.83 (16)	C43—C31—H26	119.7
C58—N2—C56	121.96 (19)	C32—C31—H26	119.7
C35—N2—C56	120.65 (18)	C33—C32—C31	118.6 (2)
C23—C1—C2	120.7 (2)	C33—C32—H29	120.7
C23—C1—H1	119.6	C31—C32—H29	120.7
C2—C1—H1	119.6	C32—C33—C58	120.4 (2)
C1—C2—C3	120.5 (2)	C32—C33—C34	130.5 (2)
C1—C2—H12	119.7	C58—C33—C34	108.93 (19)
C3—C2—H12	119.7	C33—C34—C60	115.23 (19)
C2—C3—C4	119.2 (2)	C33—C34—C44	108.60 (18)
C2—C3—C27	122.2 (2)	C60—C34—C44	109.1 (2)
C4—C3—C27	118.62 (19)	C33—C34—C35	100.53 (17)
C5—C4—C24	120.0 (2)	C60—C34—C35	112.27 (18)
C5—C4—C3	120.22 (18)	C44—C34—C35	110.90 (18)
C24—C4—C3	119.7 (2)	N2—C35—O2	106.71 (16)
C4—C5—C6	119.32 (19)	N2—C35—C47	112.72 (17)
C4—C5—C22	120.34 (18)	O2—C35—C47	112.11 (17)
C6—C5—C22	120.33 (19)	N2—C35—C34	103.49 (17)
C7—C6—C5	118.31 (19)	O2—C35—C34	106.80 (16)
C7—C6—C28	121.95 (18)	C47—C35—C34	114.32 (18)
C5—C6—C28	119.72 (19)	O2—C36—C37	116.57 (18)
O1—C7—C6	116.60 (18)	O2—C36—C45	121.74 (19)
O1—C7—C20	121.56 (19)	C37—C36—C45	121.66 (18)
C6—C7—C20	121.81 (19)	C36—C37—C38	118.41 (19)
N1—C8—O1	105.06 (17)	C36—C37—C54	122.64 (18)
N1—C8—C18	114.76 (17)	C38—C37—C54	118.92 (19)
O1—C8—C18	110.45 (17)	C49—C38—C39	120.35 (19)
N1—C8—C9	102.94 (17)	C49—C38—C37	119.94 (19)
O1—C8—C9	108.56 (16)	C39—C38—C37	119.69 (19)
C18—C8—C9	114.37 (19)	C40—C39—C38	120.39 (18)
C10—C9—C29	115.04 (19)	C40—C39—C52	120.1 (2)
C10—C9—C30	108.95 (18)	C38—C39—C52	119.5 (2)
C29—C9—C30	108.6 (2)	C41—C40—C39	119.2 (2)
C10—C9—C8	100.53 (17)	C41—C40—C53	122.3 (2)
C29—C9—C8	112.92 (18)	C39—C40—C53	118.6 (2)
C30—C9—C8	110.63 (18)	C42—C41—C40	120.3 (3)
C11—C10—C15	120.1 (2)	C42—C41—H39	119.8
C11—C10—C9	131.0 (2)	C40—C41—H39	119.8
C15—C10—C9	108.83 (19)	C55—C42—C41	121.0 (2)
C10—C11—C12	119.2 (2)	C55—C42—H27	119.5
C10—C11—H25	120.4	C41—C42—H27	119.5
C12—C11—H25	120.4	C31—C43—C59	121.2 (2)
C13—C12—C11	120.2 (2)	C31—C43—H28	119.4
C13—C12—H2	119.9	C59—C43—H28	119.4
C11—C12—H2	119.9	C34—C44—H31	109.5
C12—C13—C14	121.3 (2)	C34—C44—H30	109.5
C12—C13—H3	119.3	H31—C44—H30	109.5
C14—C13—H3	119.3	C34—C44—H32	109.5
C15—C14—C13	118.0 (2)	H31—C44—H32	109.5
C15—C14—H4	121.0	H30—C44—H32	109.5
C13—C14—H4	121.0	C48—C45—C36	119.0 (2)
C14—C15—N1	129.3 (2)	C48—C45—C46	123.4 (2)
C14—C15—C10	121.2 (2)	C36—C45—C46	117.47 (19)
N1—C15—C10	109.43 (19)	C47—C46—C45	121.6 (2)
N1—C16—C17	115.9 (2)	C47—C46—H33	119.2
N1—C16—H5	108.3	C45—C46—H33	119.2
C17—C16—H5	108.3	C46—C47—C35	123.2 (2)
N1—C16—H6	108.3	C46—C47—H34	118.4
C17—C16—H6	108.3	C35—C47—H34	118.4
H5—C16—H6	107.4	C45—C48—C49	121.5 (2)
C16—C17—H8	109.5	C45—C48—H35	119.2
C16—C17—H9	109.5	C49—C48—H35	119.2
H8—C17—H9	109.5	C48—C49—C38	119.34 (19)
C16—C17—H7	109.5	C48—C49—C50	122.3 (2)
H8—C17—H7	109.5	C38—C49—C50	118.3 (2)
H9—C17—H7	109.5	C51—C50—C49	121.4 (2)
C19—C18—C8	121.8 (2)	C51—C50—H41	119.3
C19—C18—H18	119.1	C49—C50—H41	119.3
C8—C18—H18	119.1	C50—C51—C52	121.9 (2)
C18—C19—C20	121.4 (2)	C50—C51—H40	119.0
C18—C19—H10	119.3	C52—C51—H40	119.0
C20—C19—H10	119.3	C55—C52—C51	123.2 (2)
C21—C20—C7	118.8 (2)	C55—C52—C39	118.2 (2)
C21—C20—C19	124.2 (2)	C51—C52—C39	118.5 (2)
C7—C20—C19	116.95 (19)	C54—C53—C40	121.5 (2)
C22—C21—C20	121.9 (2)	C54—C53—H36	119.3
C22—C21—H11	119.1	C40—C53—H36	119.3
C20—C21—H11	119.1	C53—C54—C37	120.93 (19)
C21—C22—C5	118.83 (19)	C53—C54—H37	119.5
C21—C22—C26	123.5 (2)	C37—C54—H37	119.5
C5—C22—C26	117.6 (2)	C42—C55—C52	121.2 (2)
C1—C23—C24	121.4 (2)	C42—C55—H38	119.4
C1—C23—H15	119.3	C52—C55—H38	119.4
C24—C23—H15	119.3	N2—C56—C57	115.63 (19)
C23—C24—C25	123.1 (2)	N2—C56—H43	108.4
C23—C24—C4	118.4 (2)	C57—C56—H43	108.4
C25—C24—C4	118.4 (2)	N2—C56—H42	108.4
C26—C25—C24	121.5 (2)	C57—C56—H42	108.4
C26—C25—H13	119.2	H43—C56—H42	107.4
C24—C25—H13	119.2	C56—C57—H46	109.5
C25—C26—C22	122.0 (2)	C56—C57—H45	109.5
C25—C26—H14	119.0	H46—C57—H45	109.5
C22—C26—H14	119.0	C56—C57—H44	109.5
C28—C27—C3	121.7 (2)	H46—C57—H44	109.5
C28—C27—H16	119.2	H45—C57—H44	109.5
C3—C27—H16	119.2	C59—C58—C33	121.6 (2)
C27—C28—C6	120.45 (19)	C59—C58—N2	128.4 (2)
C27—C28—H17	119.8	C33—C58—N2	109.94 (19)
C6—C28—H17	119.8	C58—C59—C43	117.6 (2)
C9—C29—H21	109.5	C58—C59—H47	121.2
C9—C29—H20	109.5	C43—C59—H47	121.2
H21—C29—H20	109.5	C34—C60—H50	109.5
C9—C29—H19	109.5	C34—C60—H49	109.5
H21—C29—H19	109.5	H50—C60—H49	109.5
H20—C29—H19	109.5	C34—C60—H48	109.5
C9—C30—H22	109.5	H50—C60—H48	109.5
C9—C30—H24	109.5	H49—C60—H48	109.5
			
C23—C1—C2—C3	−0.7 (3)	C43—C31—C32—C33	−0.4 (4)
C1—C2—C3—C4	1.3 (3)	C31—C32—C33—C58	−0.4 (3)
C1—C2—C3—C27	−177.4 (2)	C31—C32—C33—C34	174.4 (2)
C2—C3—C4—C5	−179.70 (18)	C32—C33—C34—C60	45.0 (3)
C27—C3—C4—C5	−0.9 (3)	C58—C33—C34—C60	−139.7 (2)
C2—C3—C4—C24	−1.7 (3)	C32—C33—C34—C44	−77.6 (3)
C27—C3—C4—C24	177.13 (18)	C58—C33—C34—C44	97.7 (2)
C24—C4—C5—C6	−177.70 (17)	C32—C33—C34—C35	166.0 (2)
C3—C4—C5—C6	0.3 (3)	C58—C33—C34—C35	−18.8 (2)
C24—C4—C5—C22	0.8 (3)	C58—N2—C35—O2	85.09 (19)
C3—C4—C5—C22	178.85 (17)	C56—N2—C35—O2	−63.4 (2)
C4—C5—C6—C7	178.80 (17)	C58—N2—C35—C47	−151.43 (18)
C22—C5—C6—C7	0.3 (3)	C56—N2—C35—C47	60.1 (3)
C4—C5—C6—C28	0.4 (3)	C58—N2—C35—C34	−27.4 (2)
C22—C5—C6—C28	−178.07 (18)	C56—N2—C35—C34	−175.84 (18)
C8—O1—C7—C6	163.87 (17)	C36—O2—C35—N2	130.42 (19)
C8—O1—C7—C20	−18.0 (3)	C36—O2—C35—C47	6.6 (3)
C5—C6—C7—O1	179.85 (17)	C36—O2—C35—C34	−119.4 (2)
C28—C6—C7—O1	−1.8 (3)	C33—C34—C35—N2	27.23 (19)
C5—C6—C7—C20	1.8 (3)	C60—C34—C35—N2	150.2 (2)
C28—C6—C7—C20	−179.91 (19)	C44—C34—C35—N2	−87.5 (2)
C15—N1—C8—O1	82.8 (2)	C33—C34—C35—O2	−85.19 (18)
C16—N1—C8—O1	−65.6 (2)	C60—C34—C35—O2	37.8 (2)
C15—N1—C8—C18	−155.68 (19)	C44—C34—C35—O2	160.09 (17)
C16—N1—C8—C18	55.9 (3)	C33—C34—C35—C47	150.21 (18)
C15—N1—C8—C9	−30.8 (2)	C60—C34—C35—C47	−86.8 (2)
C16—N1—C8—C9	−179.19 (19)	C44—C34—C35—C47	35.5 (3)
C7—O1—C8—N1	154.17 (16)	C35—O2—C36—C37	−177.71 (17)
C7—O1—C8—C18	29.9 (2)	C35—O2—C36—C45	0.4 (3)
C7—O1—C8—C9	−96.3 (2)	O2—C36—C37—C38	−179.05 (17)
N1—C8—C9—C10	29.6 (2)	C45—C36—C37—C38	2.8 (3)
O1—C8—C9—C10	−81.43 (19)	O2—C36—C37—C54	2.7 (3)
C18—C8—C9—C10	154.74 (17)	C45—C36—C37—C54	−175.46 (19)
N1—C8—C9—C29	152.7 (2)	C36—C37—C38—C49	−0.3 (3)
O1—C8—C9—C29	41.7 (3)	C54—C37—C38—C49	177.98 (18)
C18—C8—C9—C29	−82.2 (2)	C36—C37—C38—C39	−178.57 (17)
N1—C8—C9—C30	−85.4 (2)	C54—C37—C38—C39	−0.2 (3)
O1—C8—C9—C30	163.55 (17)	C49—C38—C39—C40	−177.59 (18)
C18—C8—C9—C30	39.7 (2)	C37—C38—C39—C40	0.6 (3)
C29—C9—C10—C11	42.5 (3)	C49—C38—C39—C52	1.0 (3)
C30—C9—C10—C11	−79.6 (3)	C37—C38—C39—C52	179.18 (17)
C8—C9—C10—C11	164.1 (2)	C38—C39—C40—C41	178.42 (19)
C29—C9—C10—C15	−141.0 (2)	C52—C39—C40—C41	−0.1 (3)
C30—C9—C10—C15	96.9 (2)	C38—C39—C40—C53	−0.3 (3)
C8—C9—C10—C15	−19.4 (2)	C52—C39—C40—C53	−178.88 (18)
C15—C10—C11—C12	−0.4 (3)	C39—C40—C41—C42	0.9 (3)
C9—C10—C11—C12	175.8 (2)	C53—C40—C41—C42	179.6 (2)
C10—C11—C12—C13	−0.9 (3)	C40—C41—C42—C55	−0.9 (4)
C11—C12—C13—C14	1.0 (4)	C32—C31—C43—C59	0.7 (4)
C12—C13—C14—C15	0.1 (3)	O2—C36—C45—C48	178.58 (19)
C13—C14—C15—N1	−177.6 (2)	C37—C36—C45—C48	−3.4 (3)
C13—C14—C15—C10	−1.3 (3)	O2—C36—C45—C46	−5.5 (3)
C8—N1—C15—C14	−164.1 (2)	C37—C36—C45—C46	172.55 (19)
C16—N1—C15—C14	−15.8 (3)	C48—C45—C46—C47	178.5 (2)
C8—N1—C15—C10	19.3 (2)	C36—C45—C46—C47	2.8 (3)
C16—N1—C15—C10	167.61 (18)	C45—C46—C47—C35	4.9 (3)
C11—C10—C15—C14	1.5 (3)	N2—C35—C47—C46	−129.7 (2)
C9—C10—C15—C14	−175.5 (2)	O2—C35—C47—C46	−9.3 (3)
C11—C10—C15—N1	178.42 (19)	C34—C35—C47—C46	112.5 (2)
C9—C10—C15—N1	1.5 (2)	C36—C45—C48—C49	1.4 (3)
C15—N1—C16—C17	−62.1 (3)	C46—C45—C48—C49	−174.2 (2)
C8—N1—C16—C17	82.0 (3)	C45—C48—C49—C38	1.0 (3)
N1—C8—C18—C19	−143.1 (2)	C45—C48—C49—C50	178.8 (2)
O1—C8—C18—C19	−24.6 (3)	C39—C38—C49—C48	176.70 (19)
C9—C8—C18—C19	98.2 (3)	C37—C38—C49—C48	−1.5 (3)
C8—C18—C19—C20	7.3 (3)	C39—C38—C49—C50	−1.3 (3)
O1—C7—C20—C21	179.76 (19)	C37—C38—C49—C50	−179.47 (19)
C6—C7—C20—C21	−2.3 (3)	C48—C49—C50—C51	−176.9 (2)
O1—C7—C20—C19	−2.0 (3)	C38—C49—C50—C51	1.0 (3)
C6—C7—C20—C19	175.96 (19)	C49—C50—C51—C52	−0.4 (4)
C18—C19—C20—C21	−174.6 (2)	C50—C51—C52—C55	178.8 (2)
C18—C19—C20—C7	7.3 (3)	C50—C51—C52—C39	0.1 (3)
C7—C20—C21—C22	0.6 (3)	C40—C39—C52—C55	−0.6 (3)
C19—C20—C21—C22	−177.4 (2)	C38—C39—C52—C55	−179.14 (19)
C20—C21—C22—C5	1.4 (3)	C40—C39—C52—C51	178.20 (19)
C20—C21—C22—C26	−177.87 (19)	C38—C39—C52—C51	−0.4 (3)
C4—C5—C22—C21	179.68 (18)	C41—C40—C53—C54	−179.1 (2)
C6—C5—C22—C21	−1.8 (3)	C39—C40—C53—C54	−0.4 (3)
C4—C5—C22—C26	−1.0 (3)	C40—C53—C54—C37	0.8 (3)
C6—C5—C22—C26	177.46 (18)	C36—C37—C54—C53	177.79 (19)
C2—C1—C23—C24	0.5 (3)	C38—C37—C54—C53	−0.5 (3)
C1—C23—C24—C25	178.3 (2)	C41—C42—C55—C52	0.2 (4)
C1—C23—C24—C4	−0.8 (3)	C51—C52—C55—C42	−178.2 (2)
C5—C4—C24—C23	179.43 (19)	C39—C52—C55—C42	0.5 (3)
C3—C4—C24—C23	1.4 (3)	C58—N2—C56—C57	−62.7 (3)
C5—C4—C24—C25	0.3 (3)	C35—N2—C56—C57	81.6 (3)
C3—C4—C24—C25	−177.73 (18)	C32—C33—C58—C59	0.9 (3)
C23—C24—C25—C26	179.7 (2)	C34—C33—C58—C59	−174.9 (2)
C4—C24—C25—C26	−1.2 (3)	C32—C33—C58—N2	178.92 (19)
C24—C25—C26—C22	1.0 (3)	C34—C33—C58—N2	3.1 (2)
C21—C22—C26—C25	179.4 (2)	C35—N2—C58—C59	−166.0 (2)
C5—C22—C26—C25	0.1 (3)	C56—N2—C58—C59	−18.0 (3)
C2—C3—C27—C28	179.5 (2)	C35—N2—C58—C33	16.2 (2)
C4—C3—C27—C28	0.7 (3)	C56—N2—C58—C33	164.11 (19)
C3—C27—C28—C6	0.1 (3)	C33—C58—C59—C43	−0.6 (3)
C7—C6—C28—C27	−178.94 (19)	N2—C58—C59—C43	−178.2 (2)
C5—C6—C28—C27	−0.6 (3)	C31—C43—C59—C58	−0.2 (4)

### 1-Ethyl-3,3-dimethylspiro[indoline-2,8'-phenaleno[1,9-fg]chromene] Hydrogen-bond geometry (Å, º)

*Cg*3, *Cg*6, *Cg*7, *Cg*29 and *Cg*31 are the centroids of the C1–C4/C24/C23, C5–C7/C20–C22, C10–C15, C36–C38/C45/C48/C49 and C38/C39/C49–C52 rings, respectively.

**Table:**

*D*—H···*A*	*D*—H	H···*A*	*D*···*A*	*D*—H···*A*
C16—H5···*Cg*6^i^	0.99	2.91	3.683 (3)	136
C2—H12···*Cg*3^ii^	0.95	2.86	3.698 (3)	148
C48—H35···*Cg*7	0.95	2.80	3.641 (3)	148
C56—H42···*Cg*29^iii^	0.99	2.96	3.856 (2)	151
C59—H47···*Cg*31^iii^	0.95	2.98	3.800 (3)	146

Symmetry codes: (i) −*x*+3/2, −*y*+1/2, *z*; (ii) −*x*+1, −*y*+1/2, *z*−1/2; (iii) *x*−1/4, −*y*+3/4, *z*+1/4.
